# Radiomics-Based Machine Learning with Natural Gradient Boosting for Continuous Survival Prediction in Glioblastoma

**DOI:** 10.3390/cancers16213614

**Published:** 2024-10-26

**Authors:** Mert Karabacak, Shiv Patil, Zachary Charles Gersey, Ricardo Jorge Komotar, Konstantinos Margetis

**Affiliations:** 1 Department of Neurosurgery, Mount Sinai Health System, New York, NY 10029, USA; mert.karabacak@mountsinai.org; 2 Sidney Kimmel Medical College, Thomas Jefferson University, Philadelphia, PA 19107, USA; shiv.patil@students.jefferson.edu; 3 Department of Neurological Surgery, Miller School of Medicine, University of Miami, Miami, FL 33136, USA; zgersey@med.miami.edu (Z.C.G.); rkomotar@med.miami.edu (R.J.K.)

**Keywords:** glioblastoma, survival, radiomics, machine learning, MRI

## Abstract

(1) Background: Glioblastoma (GBM) is the most common primary malignant brain tumor in adults, with an aggressive disease course that requires accurate prognosis for individualized treatment planning. This study aims to develop and evaluate a radiomics-based machine learning (ML) model to estimate overall survival (OS) for patients with GBM using pre-treatment multi-parametric magnetic resonance imaging (MRI). (2) Methods: The MRI data of 865 patients with GBM were assessed, comprising 499 patients from the UPENN-GBM dataset and 366 patients from the UCSF-PDGM dataset. A total of 14,598 radiomic features were extracted from T1, T1 with contrast, T2, and FLAIR MRI sequences using PyRadiomics. The UPENN-GBM dataset was used for model development (70%) and internal validation (30%), while the UCSF-PDGM dataset served as an external test set. The NGBoost Survival model was developed to generate continuous probability estimates as well as predictions for 6-, 12-, 18-, and 24-month OS. (3) Results: The NGBoost Survival model successfully predicted survival, achieving a C-index of 0.801 on internal validation and 0.725 on external validation. For 6-month OS, the model attained an AUROC of 0.791 (95% CI: 0.742–0.832) and 0.708 (95% CI: 0.654–0.748) for internal and external validation, respectively. (4) Conclusions: The radiomics-based ML model demonstrates potential to improve the prediction of OS for patients with GBM.

## Simple Summary

Glioblastoma is the most aggressive type of brain cancer, with patients typically surviving less than 15 months after diagnosis. Accurate prediction of survival time is crucial for tailoring treatment plans to individual patients. This study develops a new computer-based method that analyzes brain scans to forecast survival in glioblastoma patients. By examining detailed features from magnetic resonance imaging (MRI) scans, our approach aims to provide more precise and personalized survival estimates than current methods. We tested our model on a large group of patients from two different hospitals, demonstrating its ability to predict survival accurately at various time points. If validated in future studies, this tool could help doctors make more informed decisions about patient care, potentially improving outcomes for those diagnosed with this challenging disease.

## 1. Introduction

Glioblastoma (GBM) is the most common primary malignant brain tumor in adults, accounting for approximately 50% of all primary malignant central nervous system (CNS) tumors [1]. GBM is characterized by an aggressive disease course, with a median overall survival (OS) of less than 15 months [2,3]. Despite maximal treatment, including surgical resection followed by radiotherapy with concurrent temozolomide, the prognosis remains poor due to significant intra-tumor heterogeneity and treatment resistance [2,4,5]. Given the rapid progression of GBM, accurate and personalized prognostication is crucial to avoid over- or under-treatment [6]. Currently, clinicians rely on various prognostic factors, including age, Karnofsky Performance Status, and extent of surgical resection, to estimate survival in patients with GBM [7,8]. However, these generalized variables have a limited ability to capture the complexity of individual GBM cases and, therefore, may yield inaccurate prognoses [9].

Magnetic resonance imaging (MRI) is the gold standard for diagnosis and routine management of patients with GBM [10]. Semantic features derived from MRI, such as the degree of contrast enhancement, extent of peritumoral edema, and T2-weighted signal intensity, have been associated with survival outcomes in GBM patients [11]. The prognostic potential of MRI has been further expanded by recent advancements in radiomics, which enables the extraction of high-throughput, agnostic features from radiographic images that are not readily detectable by human perception alone [12]. Radiomics-based signatures can reflect the underlying pathophysiology of lesions and thus hold prognostic value, particularly when combined with machine learning (ML) models capable of automatically identifying patterns within these features [13].

A growing body of research has investigated the utility of radiomics-based models for predicting survival in oncology, including GBM [14]. These models have extracted radiomic features from pre-treatment multi-parametric MRI (mpMRI) to estimate OS and progression-free survival (PFS) in GBM patients [15,16,17,18,19,20]. However, most models have employed binary classification of OS (i.e., long vs. short survival), which limits the interpretability and clinical applicability of their results. Furthermore, the generalizability of several published models has been constrained by a lack of external validation using independent datasets [15]. To address these limitations, this study aims to develop and evaluate a radiomics-based ML model that provides continuous probability estimates of OS in patients with GBM. The model utilizes a large sample of pre-treatment mpMRI data and undergoes validation with an external cohort.

## 2. Materials and Methods

### 2.1. Data Source and Study Population

This study utilized two publicly available datasets: the University of Pennsylvania Health System Glioblastoma (UPENN-GBM) dataset [21] and the University of California San Francisco Preoperative Diffuse Glioma MRI (UCSF-PDGM) dataset [22], both accessed through The Cancer Imaging Archive (TCIA) [23]. These datasets comprise mpMRI images with the corresponding 3D segmentation masks and accompanying clinical information.

The UPENN-GBM dataset consists of pre-treatment MRI scans from 630 patients diagnosed with GBM, sourced from the University of Pennsylvania Health System [21]. The UCSF-PDGM dataset includes preoperative MRI scans from 500 adult patients with histopathologically confirmed diffuse gliomas treated at a single medical center between 2015 and 2021 [22].

For both datasets, the inclusion criteria encompassed a confirmed diagnosis of IDH wiltype, WHO grade 4 GBM, and the availability of pre-treatment scans with standard MRI sequences: pre-contrast T1-weighted (T1W), post-contrast T1-weighted (T1C), T2-weighted (T2W), and T2-weighted Fluid Attenuated Inversion Recovery (FLAIR). Patients without survival information were excluded from both datasets. Additionally, non-GBM patients were excluded from the UCSF-PDGM dataset.

The datasets provided pre-processed images and segmentation masks, with detailed processing information available elsewhere [21,22]. For both datasets, the segmentation process delineated three distinct tumor sub-regions: contrast-enhancing tumor, necrotic tumor core, and peritumoral edema.

### 2.2. Outcome of Interest and Study Design

The primary outcome of interest in this study was the prediction of OS for patients with GBM. We approached this as a true survival analysis problem, utilizing an algorithm capable of handling right-censored data. Our model was designed to predict survival probabilities across a continuous time frame, rather than as a binary classification problem. Survival time was defined as the duration from the date of GBM diagnosis to either the date of death or the last follow-up date for censored patients.

### 2.3. Radiomic Feature Extraction

To analyze each patient’s mpMRI sequences (T1W, T1C, T2W, and FLAIR), we used PyRadiomics to extract an extensive set of radiomic features [24]. We created a single binary segmentation mask combining the necrotic tumor core and enhancing tumor areas, which represented the entire lesion, excluding peritumoral edema. Image preprocessing was performed using the PyRadiomics library’s standard settings file, which includes intensity normalization and isotropic resampling to a 1 × 1 × 1 mm^3^ grid.

Our extraction process yielded 14,598 radiomic features per patient, including both original and wavelet-transformed features. These were organized into eleven distinct categories. We applied wavelet transformations using various combinations of low-pass (L) and high-pass (H) filters, such as LLL, HHH, LLH, LHL, HLL, HHL, and HLH. For each feature category, we generated five types of feature matrices: first-order statistics (18 features), Gray Level Co-occurrence Matrix (GLCM, 24 features), Gray Level Size Zone Matrix (GLSZM, 16 features), Gray Level Run Length Matrix (GLRLM, 16 features), and Gray Level Dependence Matrix (GLDM, 14 features). We also included a shape matrix (14 features) in the original feature category. This comprehensive set of features captures a broad range of image characteristics, including intensity patterns, textures, shapes, and wavelet transformations [24].

### 2.4. Data Splitting and Preprocessing

We utilized the UPENN-GBM dataset for model training and internal validation, while reserving the UCSF-PDGM dataset for external testing. The UPENN-GBM dataset was split into training (70%) and validation (30%) sets. The training set was used for feature preprocessing, dimensionality reduction, and model training, while the validation set was used for hyperparameter tuning and internal performance assessment. The UCSF-PDGM dataset served as an independent external test set for final model evaluation.

We performed standard scaling on all features. This process involved centering the features by subtracting the mean and scaling to unit variance. To prevent data leakage, we calculated the scaling parameters using only the training set and then applied these parameters to both the internal validation set and the external test set. None of the features contained missing values; thus, no imputation for missing values was applied.

### 2.5. Dimensionality Reduction

To address the high dimensionality of our radiomic feature space and reduce the risk of overfitting, we employed Principal Component Analysis (PCA) [25]. Age and sex were not included in this PCA. Using only the training set, we performed an initial PCA on the radiomic features without specifying the number of components. We then calculated the cumulative explained variance ratio and determined the number of components required to exceed our predetermined variance threshold of 0.95.

Using this optimal number of components, we fit a new PCA model on the training data. We then applied this PCA transformation to the internal validation set and the external test set. This process substantially reduced the dimensionality of our feature space, condensing the original 14,598 radiomic features into 166 principal components while retaining 95% of the variance in the data.

We then combined these principal components with the non-radiomic variables (age and sex) to create our final feature set for the survival model. Following PCA, we performed an additional round of standard scaling on the principal components. Consistent with our previous preprocessing steps, we calculated the scaling parameters using only the training data and then applied these parameters to both the internal validation and external test sets.

### 2.6. Feature Selection and Model Development

Our study employed NGBoost Survival, a probabilistic prediction algorithm that extends gradient boosting to estimate parameters of survival distributions [26]. This method is particularly well-suited for survival analysis as it handles right-censored data and provides continuous survival probability estimates.

Our approach to feature selection and model development for NGBoost Survival involved a two-stage process: initial feature ranking using Random Survival Forest (RSF) [27], followed by hyperparameter optimization and final feature selection using Optuna [28].

We first used an RSF model to rank features by importance. This process began by converting our survival data into a structured array format, combining event indicators and survival times. We fit an RSF model with 100 estimators, setting minimum samples for split and leaf nodes to 5, and used a fixed random state for reproducibility. After fitting the RSF model on the training data, we used permutation importance to evaluate feature importance on the validation set, performing this permutation five times for each feature to ensure robust estimates. Features were then ranked based on their mean importance scores.

We then used Optuna, a hyperparameter optimization framework, to simultaneously tune the NGBoost Survival model parameters and select the optimal number of features. Our objective function for optimization was designed to maximize the concordance index (C-index) [29,30] on the validation set. The hyperparameters we optimized included the number of estimators, learning rate, minibatch fraction, and column sampling rate. We also included the number of features to use as a hyperparameter, allowing Optuna to select between 1 and the total number of available features.

For each trial, we fit an NGBoost Survival model using the LogNormal distribution and the suggested hyperparameters. We used early stopping with a patience of 100 rounds to prevent overfitting. We ran 100 optimization trials, allowing Optuna to explore the hyperparameter space and find the combination that yielded the highest C-index.

Using the optimal hyperparameters and the number of features determined by Optuna, we trained the final NGBoost Survival model. This comprehensive approach allowed us to leverage the strengths of both Random Survival Forest for initial feature ranking and Optuna for fine-tuned hyperparameter optimization and feature selection, resulting in an optimized NGBoost Survival model for our task.

### 2.7. Model Evaluation

We evaluated our NGBoost Survival model’s performance using both continuous survival metrics and time-dependent binary classification metrics. This evaluation was conducted on two distinct datasets: an internal validation set (30% of the UPENN-GBM dataset) and an external test set (the entire UCSF-PDGM dataset).

For continuous survival prediction, we used the C-index as our primary metric. For time-dependent evaluations, we calculated performance metrics for predicting survival at 6, 12, 18, and 24 months. These metrics included precision, recall, F1 score, accuracy, Matthews Correlation Coefficient (MCC), area under the receiver operating characteristic curve (AUROC), area under the precision–recall curve (AUPRC), and Brier score.

To account for varying follow-up times, we employed a specific approach for these time-dependent evaluations. For each time point (6, 12, 18, and 24 months), we excluded patients with follow-up times shorter than the evaluation period and no recorded event. This ensured that the evaluation only included patients with sufficient follow-up or a recorded event within the time frame of interest.

For these binary classifications, we utilized the Youden index to determine the optimal classification threshold at each time point. This threshold was identified as the point on the receiver operating characteristic (ROC) curve corresponding to the maximum value of the Youden Index (J = sensitivity + specificity − 1) [31,32]. We calculated 95% confidence intervals (CIs) for all time-dependent metrics using bootstrap resampling with 1000 iterations.

To aid in model interpretation and visualization, we generated ROC curves and precision–recall curves (PRCs) for both the internal validation set and the external test set at each evaluated time point (6, 12, 18, and 24 months).

## 3. Results

Our final dataset consisted of 865 patients with GBM, comprising 499 patients from the UPENN-GBM dataset and 366 patients from the UCSF-PDGM dataset. The UPENN-GBM dataset was used for model development and internal validation, while the UCSF-PDGM dataset served as an external test set.

In the UPENN-GBM cohort, the median age was 63 years (IQR: 15), and 59.7% of the patients were male. The median overall survival was 404 days. In the UCSF-PDGM cohort, the median age was 57 years (IQR: 21), and 59.8% of the patients were male. The median overall survival for this cohort was 509 days.

The NGBoost Survival model demonstrated robust predictive capability for survival outcomes in patients with GBM. For continuous survival prediction, the model achieved a C-index of 0.801 on the internal validation set and 0.725 on the external test set, indicating good discriminative ability across both cohorts.

Time-dependent performance metrics revealed strong predictive power across different time horizons, as shown in Table 1. For the internal validation set, the AUROC at 6, 12, 18, and 24 months was 0.791 (95% CI: 0.742–0.832), 0.780 (95% CI: 0.740–0.821), 0.806 (95% CI: 0.764–0.844), and 0.815 (95% CI: 0.760–0.861), respectively. The corresponding AUROCs for the external test set were 0.708 (95% CI: 0.654–0.748), 0.702 (95% CI: 0.661–0.741), 0.714 (95% CI: 0.675–0.753), and 0.657 (95% CI: 0.606–0.700).

**Figure 1:**
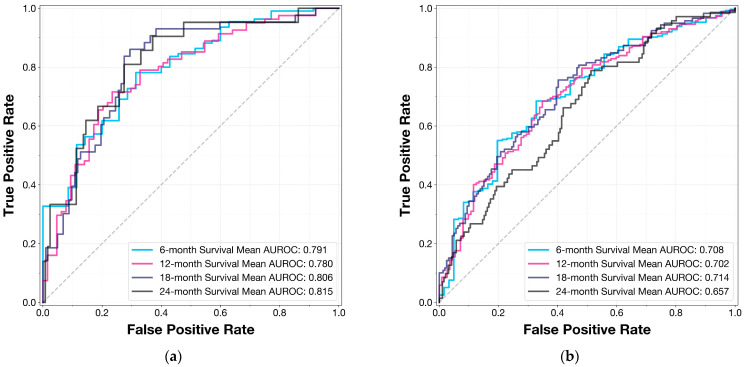
Models’ receiver operating characteristics curves for time-dependent evaluations for the (**a**) internal validation set and (**b**) external test set (AUROC: area under receiver operating characteristics curve).

Figure 1 illustrates the receiver operating characteristic (ROC) curves for both the internal validation set (Figure 1a) and the external test set (Figure 1b) at all evaluated time points. These curves visually demonstrate the model’s discriminative ability across different survival thresholds and patient populations.

**Figure 2:**
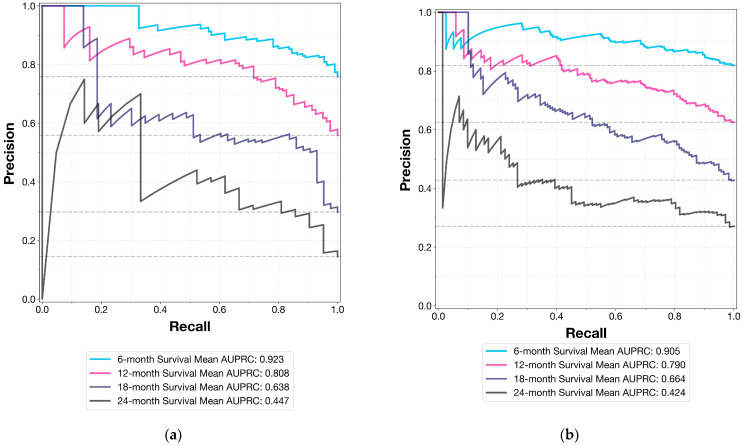
Models’ precision–recall curves for time-dependent evaluations (**a**) for the internal validation set and (**b**) external test set (AUPRC: area under receiver operating characteristics curve).

Figure 2 presents the precision–recall curves for the internal validation set (Figure 2a) and the external test set (Figure 2b) at all evaluated time points. These curves provide insight into the model’s performance across different classification thresholds, particularly in the context of class imbalance.

For 6-month survival prediction on the internal validation set, the model achieved a precision of 0.887 (95% CI: 0.848–0.917), recall of 0.782 (95% CI: 0.739–0.820), and F1 score of 0.831 (95% CI: 0.796–0.857). The accuracy was 0.759 (95% CI: 0.717–0.793), and the Matthews Correlation Coefficient (MCC) was 0.425 (95% CI: 0.333–0.504). The area under the precision–recall curve (AUPRC) was 0.923 (95% CI: 0.898–0.943), and the Brier score was 0.179 (95% CI: 0.163–0.196).

On the external test set, 6-month survival prediction showed a precision of 0.904 (95% CI: 0.877–0.928), recall of 0.685 (95% CI: 0.642–0.717), and F1 score of 0.779 (95% CI: 0.750–0.804). The accuracy was 0.682 (95% CI: 0.647–0.715), and the MCC was 0.283 (95% CI: 0.210–0.351). The AUPRC was 0.905 (95% CI: 0.874–0.928), and the Brier score was 0.140 (95% CI: 0.126–0.153).

Similar patterns of performance were observed for 12-, 18-, and 24-month predictions, with the model generally showing strong discriminative ability and calibration across both the internal validation and external test sets (Table 1). The optimal classification thresholds, determined using the Youden index, varied across time points and between the internal validation and external test sets, reflecting the model’s adaptation to different survival time horizons and patient populations.

## 4. Discussion

This study evaluated the performance of a radiomics-based ML approach to predict OS for patients with GBM. The model provided both continuous probability estimates and time-dependent predictions using features extracted from pre-treatment T1W, T1C, T2W, and FLAIR MRI sequences. NGBoost Survival demonstrated strong predictive performance, achieving a C-index of 0.801 on the internal validation set and 0.725 on the external validation set. For 6-month OS prediction, the model attained an AUROC of 0.791 (95% CI: 0.742–0.832) and 0.708 (95% CI: 0.654–0.748) for internal and external validation, respectively. Predictive performance for 12- and 18-month OS was similar, with AUROC values ranging from 0.657 to 0.815 across both datasets.

Several studies have assessed the utility of radiomics-based models to predict OS for patients with GBM. Chen et al. developed a classifier using radiomic features from post-contrast T1W that demonstrated superior predictive accuracy compared to a clinical risk model (AUROCs of 0.815 and 0.670, respectively) [17]. Bae et al. found that integrating radiomic features from T1W, T2W, and FLAIR sequences with clinical and genetic profiles improved the performance of a Random Survival Forest model for OS prediction (AUROC of 0.76) [15]. A separate model by Park et al. also exhibited improved accuracy when radiomic features from mpMRI (T1C, FLAIR, diffusion-weighted imaging, and dynamic susceptibility contrast imaging) were combined with clinical factors (C-index of 0.70 on external validation) [33]. Overall, the model evaluated in the present study demonstrated similar accuracy and performance metrics to those reported in the literature.

To date, most prognostic radiomic models of GBM have approached survival prediction as a task of binary classification, providing a likelihood estimate that a patient has “long” or “short” survival based on median OS [34]. This method of categorization has several limitations, including significant susceptibility to the survival outcomes of the particular study cohort on which the model is trained, as well as a restricted ability to provide accurate, individualized prognoses for patients [35]. Our study addressed these limitations by generating continuous probability estimates using the NGBoost model, which has previously been shown to accurately predict survival for other cancers [36,37]. This approach enables the prediction of survival at any time point for patients, irrespective of median OS at the group level. It affords a more nuanced understanding of patient prognosis and accounts for censored data points, which are often neglected by models that utilize categorical time intervals [38]. Additionally, our model is capable of predicting discrete 6-, 12-, and 18-month OS, which may be useful in scenarios where distributional assumptions for continuous-time survival prediction models are not met [35].

To our knowledge, this study utilized the largest sample of patients ever analyzed in the literature on radiomics-based survival prediction for GBM [34]. A key strength of our approach was the inclusion of an external validation set: the use of a large sample size obtained from two different institutions reduces the risk of overfitting and strengthens the generalizability of our model. Training a radiomics-based model on representative external data is critical to account for real-world differences in institutional populations and image acquisition [39]. The consistent performance we obtained between internal and external datasets highlights the robustness and stability of our model. Our findings contribute to a growing body of evidence indicating the potential of radiomics-based ML models to improve prognostication for patients with GBM.

The calculation of an accurate and personalized prognosis is fundamental to making informed clinical decisions in the management of patients with GBM. Despite the vast heterogeneity of survival outcomes for this tumor, current methods of assessing prognosis largely depend on clinical factors that are non-specific to the lesion (e.g., age, Karnofsky Performance Status, and extent of surgical resection) [7,40]. Radiomic features extracted from pre-treatment neuroimaging can reveal tumor characteristics pertinent to survival, with each imaging sequence providing unique insight into the tumor microenvironment [15,34]. For instance, texture features derived from T1W reflect tumor heterogeneity and have been shown to contribute prognostic value in GBM [17]. Texture features from non-enhancing T2 hyperintense lesions, corresponding to the peritumoral region—a site of infiltrating tumor cells and vasogenic edema—may describe the aggressiveness of disease [41,42]. The integration of tumor-specific radiomic features with conventional clinical risk algorithms presents a promising opportunity to improve prognostic accuracy for individual patients with GBM, enabling more informed decision-making to optimize treatment approaches.

Our study has several limitations. First, the data were analyzed retrospectively, which may introduce selection bias. Future research can mitigate this risk by implementing a prospective design. Second, we did not include clinical variables or tumor genetic information in our model, as these variables were not consistently available across both datasets. The inclusion of these parameters may further improve model performance, as demonstrated in other studies [15,33]. Third, while our model extracted features from multiple MRI sequences, future studies may benefit from the analysis of additional neuroimaging modalities. Signatures derived from other techniques, such as diffusion-weighted imaging and positron emission tomography, have demonstrated prognostic potential and may therefore enhance the capabilities of radiomics-based models [43,44].

## 5. Conclusions

In this study, a radiomics-based ML model leveraging pre-treatment mpMRI demonstrated robust predictive capability for overall survival in patients with GBM. This model, trained on a large sample size and validated with an external dataset, supports generalizable performance for GBM survival assessment. An additional advantage of our model is its ability to provide both continuous probability estimates and predictions for 6-, 12-, 18-, and 24-month survival. The observed findings present a promising advancement in the individualized assessment of GBM survival. Future prospective studies are required to validate the practical integration of radiomics-based models in the clinical setting.

## Figures and Tables

**Table 1:** Time-dependent evaluation metrics of the model for the internal validation and external test sets (CI, confidence interval; MCC, Matthews Correlation Coefficient; AUROC, the area under the receiver operating characteristic curve; AUPRC, the area under the precision–recall curve).

	Evaluation	Precision	Recall	F1 Score	Accuracy	MCC	AUROC	AUPRC	Brier Score
	Point	(95% CI)	(95% CI)	(95% CI)	(95% CI)	(95% CI)	(95% CI)	(95% CI)	(95% CI)
**Internal Validation Set**	**6-Month**	0.887	0.782	0.831	0.759	0.425	0.791	0.923	0.179
	**Survival**	(0.848–0.917)	(0.739–0.820)	(0.796–0.857)	(0.717–0.793)	(0.333–0.504)	(0.742–0.832)	(0.898–0.943)	(0.163–0.196)
	**12-Month**	0.795	0.716	0.753	0.738	0.478	0.780	0.808	0.248
	**Survival**	(0.735–0.840)	(0.662–0.764)	(0.711–0.790)	(0.697–0.772)	(0.400–0.548)	(0.740–0.821)	(0.758–0.850)	(0.246–0.250)
	**18-Month**	0.562	0.837	0.673	0.759	0.518	0.806	0.638	0.206
	**Survival**	(0.508–0.635)	(0.778–0.894)	(0.624–0.732)	(0.724–0.800)	(0.460–0.601)	(0.764–0.844)	(0.559–0.707)	(0.194–0.221)
	**24-Month**	0.333	0.810	0.472	0.738	0.395	0.815	0.447	0.129
	**Survival**	(0.268–0.411)	(0.700–0.889)	(0.394–0.549)	(0.703–0.779)	(0.311–0.476)	(0.760–0.861)	(0.312–0.523)	(0.114–0.147)
**External Test Set**	**6-Month**	0.904	0.685	0.779	0.682	0.283	0.708	0.905	0.140
	**Survival**	(0.877–0.928)	(0.642–0.717)	(0.750–0.804)	(0.647–0.715)	(0.210–0.351)	(0.654–0.748)	(0.874–0.928)	(0.126–0.153)
	**12-Month**	0.766	0.684	0.723	0.672	0.328	0.702	0.790	0.233
	**Survival**	(0.720–0.807)	(0.640–0.729)	(0.688–0.759)	(0.635–0.706)	(0.250–0.399)	(0.661–0.741)	(0.743–0.828)	(0.226–0.241)
	**18-Month**	0.584	0.756	0.659	0.665	0.352	0.714	0.664	0.240
	**Survival**	(0.539–0.636)	(0.705–0.805)	(0.619–0.699)	(0.629–0.698)	(0.285–0.418)	(0.675–0.753)	(0.607–0.716)	(0.225–0.256)
	**24-Month**	0.370	0.662	0.475	0.603	0.216	0.657	0.424	0.195
	**Survival**	(0.318–0.422)	(0.586–0.728)	(0.423–0.529)	(0.569–0.641)	(0.139–0.284)	(0.606–0.700)	(0.338–0.484)	(0.174–0.217)

## Data Availability

Restrictions apply to the availability of these data. Data were obtained from The Cancer imaging archive and are available at https://www.cancerimagingarchive.net/collection/upenn-gbm and https://www.cancerimagingarchive.net/collection/ucsf-pdgm with the permission of The Cancer Imaging Archive. The source code for preprocessing and analyzing the data is available on GitHub (https://github.com/mertkarabacak/GBM-Radiomics-Survival).

## References

[1] Koshy M., Villano J.L., Dolecek T.A., Howard A., Mahmood U., Chmura S.J., Weichselbaum R.R., McCarthy B.J. (2012). Improved Survival Time Trends for Glioblastoma Using the SEER 17 Population-Based Registries. J. Neurooncol..

[2] Stupp R., Mason W.P., Van Den Bent M.J., Weller M., Fisher B., Taphoorn M.J.B., Belanger K., Brandes A.A., Marosi C., Bogdahn U. (2005). Radiotherapy plus Concomitant and Adjuvant Temozolomide for Glioblastoma. N. Engl. J. Med..

[3] Stupp R., Hegi M.E., Mason W.P., van den Bent M.J., Taphoorn M.J.B., Janzer R.C., Ludwin S.K., Allgeier A., Fisher B., Belanger K. (2009). Effects of Radiotherapy with Concomitant and Adjuvant Temozolomide versus Radiotherapy Alone on Survival in Glioblastoma in a Randomised Phase III Study: 5-Year Analysis of the EORTC-NCIC Trial. Lancet Oncol..

[4] García-Montaño L.A., Licón-Muñoz Y., Martinez F.J., Keddari Y.R., Ziemke M.K., Chohan M.O., Piccirillo S.G.M. (2023). Dissecting Intra-Tumor Heterogeneity in the Glioblastoma Microenvironment Using Fluorescence-Guided Multiple Sampling. Mol. Cancer Res..

[5] Aum D.J., Kim D.H., Beaumont T.L., Leuthardt E.C., Dunn G.P., Kim A.H. (2014). Molecular and Cellular Heterogeneity: The Hallmark of Glioblastoma. Neurosurg. Focus.

[6] Mauer M., Stupp R., Taphoorn M.J.B., Coens C., Osoba D., Marosi C., Wong R., De Witte O., Cairncross J.G., Efficace F. (2007). The Prognostic Value of Health-Related Quality-of-Life Data in Predicting Survival in Glioblastoma Cancer Patients: Results from an International Randomised Phase III EORTC Brain Tumour and Radiation Oncology Groups, and NCIC Clinical Trials Group Study. Br. J. Cancer.

[7] Slika H., Karimov Z., Alimonti P., Abou-Mrad T., De Fazio E., Alomari S., Tyler B. (2023). Preclinical Models and Technologies in Glioblastoma Research: Evolution, Current State, and Future Avenues. Int. J. Mol. Sci..

[8] Lamborn K.R., Chang S.M., Prados M.D. (2004). Prognostic Factors for Survival of Patients with Glioblastoma: Recursivepartitioning Analysis. Neuro-Oncology.

[9] Briceno N., Vera E., Komlodi-Pasztor E., Abdullaev Z., Choi A., Grajkowska E., Kunst T., Levine J., Lindsley M., Fernandez K. (2024). Long-Term Survivors of Glioblastoma: Tumor Molecular, Clinical, and Imaging Findings. Neuro-Oncol. Adv..

[10] Bernstock J.D., Gary S.E., Klinger N., Valdes P.A., Ibn Essayed W., Olsen H.E., Chagoya G., Elsayed G., Yamashita D., Schuss P. (2022). Standard Clinical Approaches and Emerging Modalities for Glioblastoma Imaging. Neuro-Oncol. Adv..

[11] Gevaert O., Mitchell L.A., Achrol A.S., Xu J., Echegaray S., Steinberg G.K., Cheshier S.H., Napel S., Zaharchuk G., Plevritis S.K. (2014). Glioblastoma Multiforme: Exploratory Radiogenomic Analysis by Using Quantitative Image Features. Radiology.

[12] Gillies R.J., Kinahan P.E., Hricak H. (2016). Radiomics: Images Are More than Pictures, They Are Data. Radiology.

[13] Tomaszewski M.R., Gillies R.J. (2021). The Biological Meaning of Radiomic Features. Radiology.

[14] Gore S., Chougule T., Jagtap J., Saini J., Ingalhalikar M. (2021). A Review of Radiomics and Deep Predictive Modeling in Glioma Characterization. Acad. Radiol..

[15] Bae S., Choi Y.S., Ahn S.S., Chang J.H., Kang S.-G., Kim E.H., Kim S.H., Lee S.-K. (2018). Radiomic MRI Phenotyping of Glioblastoma: Improving Survival Prediction. Radiology.

[16] Baid U., Rane S.U., Talbar S., Gupta S., Thakur M.H., Moiyadi A., Mahajan A. (2020). Overall Survival Prediction in Glioblastoma With Radiomic Features Using Machine Learning. Front. Comput. Neurosci..

[17] Chen X., Fang M., Dong D., Liu L., Xu X., Wei X., Jiang X., Qin L., Liu Z. (2019). Development and Validation of a MRI-Based Radiomics Prognostic Classifier in Patients with Primary Glioblastoma Multiforme. Acad. Radiol..

[18] Wang S., Xiao F., Sun W., Yang C., Ma C., Huang Y., Xu D., Li L., Chen J., Li H. (2022). Radiomics Analysis Based on Magnetic Resonance Imaging for Preoperative Overall Survival Prediction in Isocitrate Dehydrogenase Wild-Type Glioblastoma. Front. Neurosci..

[19] Fathi Kazerooni A., Saxena S., Toorens E., Tu D., Bashyam V., Akbari H., Mamourian E., Sako C., Koumenis C., Verginadis I. (2022). Clinical Measures, Radiomics, and Genomics Offer Synergistic Value in AI-Based Prediction of Overall Survival in Patients with Glioblastoma. Sci. Rep..

[20] Pease M., Gersey Z.C., Ak M., Elakkad A., Kotrotsou A., Zenkin S., Elshafeey N., Mamindla P., Kumar V.A., Kumar A.J. (2022). Pre-Operative MRI Radiomics Model Non-Invasively Predicts Key Genomic Markers and Survival in Glioblastoma Patients. J. Neurooncol..

[21] Bakas S., Sako C., Akbari H., Bilello M., Sotiras A., Shukla G., Rudie J.D., Santamaría N.F., Kazerooni A.F., Pati S. (2022). The University of Pennsylvania Glioblastoma (UPenn-GBM) Cohort: Advanced MRI, Clinical, Genomics, & Radiomics. Sci. Data.

[22] Calabrese E., Villanueva-Meyer J.E., Rudie J.D., Rauschecker A.M., Baid U., Bakas S., Cha S., Mongan J.T., Hess C.P. (2022). The University of California San Francisco Preoperative Diffuse Glioma MRI Dataset. Radiol. Artif. Intell..

[23] Clark K., Vendt B., Smith K., Freymann J., Kirby J., Koppel P., Moore S., Phillips S., Maffitt D., Pringle M. (2013). The Cancer Imaging Archive (TCIA): Maintaining and Operating a Public Information Repository. J. Digit. Imaging.

[24] van Griethuysen J.J.M., Fedorov A., Parmar C., Hosny A., Aucoin N., Narayan V., Beets-Tan R.G.H., Fillion-Robin J.-C., Pieper S., Aerts H.J.W.L. (2017). Computational Radiomics System to Decode the Radiographic Phenotype. Cancer Res..

[25] Jolliffe I.T., Cadima J. (2016). Principal Component Analysis: A Review and Recent Developments. Philos. Trans. R. Soc. Math. Phys. Eng. Sci..

[26] Duan T., Avati A., Ding D.Y., Thai K.K., Basu S., Ng A.Y., Schuler A. (2020). NGBoost: Natural Gradient Boosting for Probabilistic Prediction. Proc. Mach. Learn. Res..

[27] Ishwaran H., Kogalur U.B., Blackstone E.H., Lauer M.S. (2008). Random Survival Forests. Ann. Appl. Stat..

[28] Akiba T., Sano S., Yanase T., Ohta T., Koyama M. Optuna: A Next-Generation Hyperparameter Optimization Framework. Proceedings of the KDD ’19: The 25th ACM SIGKDD Conference on Knowledge Discovery and Data Mining.

[29] Hartman N., Kim S., He K., Kalbfleisch J.D. (2023). Concordance Indices with Left-Truncated and Right-Censored Data. Biometrics.

[30] Harrell F.E., Harrell F.E. (2015). Case Study in Parametric Survival Modeling and Model Approximation. Regression Modeling Strategies: With Applications to Linear Models, Logistic and Ordinal Regression, and Survival Analysis.

[31] Youden W.J. (1950). Index for Rating Diagnostic Tests. Cancer.

[32] Fluss R., Faraggi D., Reiser B. (2005). Estimation of the Youden Index and Its Associated Cutoff Point. Biom. J..

[33] Park J.E., Kim H.S., Jo Y., Yoo R.-E., Choi S.H., Nam S.J., Kim J.H. (2020). Radiomics Prognostication Model in Glioblastoma Using Diffusion- and Perfusion-Weighted MRI. Sci. Rep..

[34] Hajianfar G., Haddadi Avval A., Hosseini S.A., Nazari M., Oveisi M., Shiri I., Zaidi H. (2023). Time-to-Event Overall Survival Prediction in Glioblastoma Multiforme Patients Using Magnetic Resonance Imaging Radiomics. Radiol. Med..

[35] Sloma M., Syed F.J., Nemati M., Xu K.S. (2021). Empirical Comparison of Continuous and Discrete-Time Representations for Survival Prediction. Proc. Mach. Learn. Res..

[36] Noh B., Park Y.M., Kwon Y., Choi C.I., Choi B.K., Seo K.I., Park Y.-H., Yang K., Lee S., Ha T. (2022). Machine Learning-Based Survival Rate Prediction of Korean Hepatocellular Carcinoma Patients Using Multi-Center Data. BMC Gastroenterol..

[37] Kim J.K., Lee S., Hong S.K., Kwak C., Jeong C.W., Kang S.H., Hong S.-H., Kim Y.-J., Chung J., Hwang E.C. (2023). Machine Learning Based Prediction for Oncologic Outcomes of Renal Cell Carcinoma after Surgery Using Korean Renal Cell Carcinoma (KORCC) Database. Sci. Rep..

[38] Kim S.Y. (2023). GNN-Surv: Discrete-Time Survival Prediction Using Graph Neural Networks. Bioengineering.

[39] Ligero M., Jordi-Ollero O., Bernatowicz K., Garcia-Ruiz A., Delgado-Muñoz E., Leiva D., Mast R., Suarez C., Sala-Llonch R., Calvo N. (2021). Minimizing Acquisition-Related Radiomics Variability by Image Resampling and Batch Effect Correction to Allow for Large-Scale Data Analysis. Eur. Radiol..

[40] Parks C., Heald J., Hall G., Kamaly-Asl I. (2013). Can the Prognosis of Individual Patients with Glioblastoma Be Predicted Using an Online Calculator?. Neuro-Oncology.

[41] Kim Y., Kim K.H., Park J., Yoon H.I., Sung W. (2023). Prognosis Prediction for Glioblastoma Multiforme Patients Using Machine Learning Approaches: Development of the Clinically Applicable Model. Radiother. Oncol..

[42] Pak E., Choi K.S., Choi S.H., Park C.-K., Kim T.M., Park S.-H., Lee J.H., Lee S.-T., Hwang I., Yoo R.-E. (2021). Prediction of Prognosis in Glioblastoma Using Radiomics Features of Dynamic Contrast-Enhanced MRI. Korean J. Radiol..

[43] Wang Z., Guan F., Duan W., Guo Y., Pei D., Qiu Y., Wang M., Xing A., Liu Z., Yu B. (2023). Diffusion Tensor Imaging-based Machine Learning for IDH Wild-type Glioblastoma Stratification to Reveal the Biological Underpinning of Radiomic Features. CNS Neurosci. Ther..

[44] Li Z., Holzgreve A., Unterrainer L.M., Ruf V.C., Quach S., Bartos L.M., Suchorska B., Niyazi M., Wenter V., Herms J. (2023). Combination of Pre-Treatment Dynamic [18F]FET PET Radiomics and Conventional Clinical Parameters for the Survival Stratification in Patients with IDH-Wildtype Glioblastoma. Eur. J. Nucl. Med. Mol. Imaging.

